# Genomics Evolutionary History and Diagnostics of the *Alternaria alternata* Species Group Including Apple and Asian Pear Pathotypes

**DOI:** 10.3389/fmicb.2019.03124

**Published:** 2020-01-23

**Authors:** Andrew D. Armitage, Helen M. Cockerton, Surapareddy Sreenivasaprasad, James Woodhall, Charles R. Lane, Richard J. Harrison, John P. Clarkson

**Affiliations:** ^1^NIAB EMR, East Malling, United Kingdom; ^2^Natural Resources Institute, University of Greenwich, Chatham Maritime, London, United Kingdom; ^3^School of Life Sciences, University of Bedfordshire, Luton, United Kingdom; ^4^Parma Research and Extension Center, University of Idaho, Parma, ID, United States; ^5^FERA Science Ltd., York, United Kingdom; ^6^Warwick Crop Centre, University of Warwick, Warwick, United Kingdom

**Keywords:** CDC, host-specific toxin, mating type, Dothideomycetes, *Alternaria mali*, *Alternaria gaisen*, nanopore

## Abstract

The *Alternaria* section *alternaria* (*Alternaria alternata* species group) represents a diverse group of saprotroph, human allergens, and plant pathogens. *Alternaria* taxonomy has benefited from recent phylogenetic revision but the basis of differentiation between major phylogenetic clades within the group is not yet understood. Furthermore, genomic resources have been limited for the study of host-specific pathotypes. We report near complete genomes of the apple and Asian pear pathotypes as well as draft assemblies for a further 10 isolates representing *Alternaria tenuissima* and *Alternaria arborescens* lineages. These assemblies provide the first insights into differentiation of these taxa as well as allowing the description of effector and non-effector profiles of apple and pear conditionally dispensable chromosomes (CDCs). We define the phylogenetic relationship between the isolates sequenced in this study and a further 23 *Alternaria* spp. based on available genomes. We determine which of these genomes represent MAT1-1-1 or MAT1-2-1 idiomorphs and designate host-specific pathotypes. We show for the first time that the apple pathotype is polyphyletic, present in both the *A. arborescens* and *A. tenuissima* lineages. Furthermore, we profile a wider set of 89 isolates for both mating type idiomorphs and toxin gene markers. Mating-type distribution indicated that gene flow has occurred since the formation of *A. tenuissima* and *A. arborescens* lineages. We also developed primers designed to *AMT14*, a gene from the apple pathotype toxin gene cluster with homologs in all tested pathotypes. These primers allow identification and differentiation of apple, pear, and strawberry pathotypes, providing new tools for pathogen diagnostics.

## Introduction

Species within the genus *Alternaria* encompass a range of lifestyles, acting as saprotroph, opportunistic pathogens, and host-adapted plant pathogens (Thomma, 2003). Large spored species include *Alternaria solani*, a major pathogen of potato, whereas small spored taxa include the *Alternaria alternata* species group (*Alternaria* sect. *alternaria*), which are found ubiquitously in the environment acting as saprotrophs and opportunistic necrotrophs. This species group is responsible for opportunistic human infections and a range of host adapted plant diseases.

Taxonomy within this presumed asexual genus has been subject to recent revision (Lawrence et al., 2013). Large spored species can be clearly resolved by standard phylogenetic markers such as ITS and are supported by morphological characters. However, small spored species within the *A. alternata* species group overlap in morphological characters, possess the same ITS haplotype (Kusaba and Tsuge, 1995), and show low variation in other commonly used barcoding markers (Lawrence et al., 2013; Armitage et al., 2015; Woudenberg et al., 2015). Highly variable phylogenetic markers have provided resolution between groups of isolates that possess morphological patterns typical of descriptions for *Alternaria gaisen*, *Alternaria tenuissima*, and *Alternaria arborescens* (Armitage et al., 2015).

The taxonomy of the species group is complicated by designation of isolates as pathotypes, each able to produce polyketide host-selective toxins (HST) adapted to apple, Asian pear, tangerine, citrus, rough lemon, or tomato (Tsuge et al., 2013). Genes involved in the production of these HSTs are located on conditionally dispensable chromosomes (CDCs) (Hatta et al., 2002). CDCs have been estimated to be 1.05 Mb in the strawberry pathotype (Hatta et al., 2002), 1.1–1.7 Mb in the apple pathotype (Johnson et al., 2001), 1.1–1.9 Mb in the tangerine pathotype (Masunaka et al., 2000, 2005), and 4.1 Mb in the pear pathotype (Tanaka et al., 1999; Tanaka and Tsuge, 2000). These CDCs are understood to have been acquired through horizontal gene transfer and as such, the evolutionary history of CDCs may be distinct from the core genome.

The polyketide synthase genes responsible for the production of the six HSTs are present in clusters. Some genes within these clusters are conserved between pathotypes (Hatta et al., 2002; Miyamoto et al., 2009), while genes are also present within these clusters that are unique to particular pathotypes (Ajiro et al., 2010; Miyamoto et al., 2010). This is reflected in structural similarities between the pear (AKT) and strawberry (AFT) and tangerine (ACTT) toxins with each containing a 9,10-epoxy-8-hydroxy-9-methyl-decatrienoic acid moiety. In contrast, the toxin produced by the apple pathotype (AMT) does not contain this moiety and is primarily cyclic in structure (Tsuge et al., 2013).

Studies making use of bacterial artificial chromosomes (BAC) have led to the sequencing of toxin gene cluster regions from three apple pathotype isolates (GenBank accessions: AB525198, AB525199, AB525200; unpublished). These sequences are 100–130 kb in size and contain 17 genes that are considered to be involved in synthesis of the AMT apple toxin (Harimoto et al., 2007). *AMT1*, *AMT2*, *AMT3*, and *AMT4* have been demonstrated to be involved in AMT synthesis, as gene disruption experiments have led to loss of toxin production and pathogenicity (Johnson et al., 2000; Harimoto et al., 2007, 2008). However, experimental evidence has not been provided to show that the remaining 13 *AMT* genes have a role in toxin production. Four genes present in the CDC for the pear pathotype have been identified and have been named *AKT1*, *AKT2*, *AKT3*, *AKTR-1* (Tanaka et al., 1999; Tanaka and Tsuge, 2000; Tsuge et al., 2013) and a further two genes (*AKT4*, *AKTS1*) have been reported (Tsuge et al., 2013).

The toxicity of an HST is not restricted to the designated host for that pathotype. All or some of the derivatives of a toxin may induce necrosis on “non-target” host leaves. For example, AMT from the apple pathotype can induce necrosis on the leaves of Asian pear (Kohmoto et al., 1976). Therefore, non-host resistance may be triggered by recognition of non-HST avirulence genes.

*Alternaria* spp. are of phytosanitary importance, with apple and pear pathotypes subject to quarantine regulations in Europe under Annex IIAI of Directive 2000/29/EC as *A. alternata* (non-European pathogenic isolates). As such, rapid and accurate diagnostics are required for identification. Where genes on essential chromosomes can be identified that phylogenetically resolve taxa, then these can be used for identification of quarantine pathogens (Bonants et al., 2010; Quaedvlieg et al., 2012). However, regulation and management strategies also need to consider the potential for genetic exchange between species. The *Alternaria* sect. *alternaria* are presumed asexual but evidence has been presented for either the presence of sexuality or a recent sexual past. Sexuality or parasexuality provides a mechanism for reshuffling the core genome associated with CDCs of a pathotype. It is currently unknown whether pathotype identification can be based on sequencing of phylogenetic loci, or whether the use of CDC-specific primers is more appropriate. This is of particular importance for the apple and Asian pear pathotypes due to the phytosanitary risk posed by their potential establishment and spread in Europe.

## Materials and Methods

Twelve genomes were sequenced, selected from a collection of isolates whose phylogenetic identity was determined in previous work (Armitage et al., 2015). These 12 isolates were three *A. arborescens* clade isolates (*FERA 675*, *RGR 97.0013*, and *RGR 97.0016*), four *A. tenuissima* clade isolates (*FERA 648*, *FERA 1082*, *FERA 1164*, and *FERA 24350*), three *A. tenuissima* clade apple pathotype isolates (*FERA 635*, *FERA 743*, *FERA 1166*, and *FERA 1177*), and one *A. gaisen* clade pear pathotype isolate (*FERA 650*).

### DNA and RNA Extraction and Sequencing

Apple pathotype isolate *FERA 1166* and Asian pear pathotype isolate *FERA 650* were sequenced using both Illumina and nanopore MinION sequencing technologies and the remaining 10 isolates were sequenced using Illumina sequencing technology. For both illumina and MinION sequencing, DNA extraction was performed on freeze dried mycelium grown in PDB for 14 days.

High molecular weight DNA was extracted for MinION sequencing using the protocol of Schwessinger and McDonald (2017), scaled down to a starting volume of 2 ml. This was followed by phenol-chloroform purification and size selection to a minimum of 30 kb using a Blue Pippin. The resulting product was concentrated using ampure beads before library preparation was performed using a Rapid Barcoding Sequencing Kit (SQK-RBK001) modified through exclusion of LLB beads. Sequencing was performed on an Oxford Nanopore GridION generating 40 and 34 times coverage of sequence data for isolates *FERA 1166* and *FERA 650*, respectively.

gDNA for illumina sequencing of isolate *FERA 1166* was extracted using a modified CTAB protocol (Li et al., 1994). gDNA for illumina sequencing of the eleven other isolates was extracted using a Genelute Plant DNA Miniprep Kit (Sigma) using the manufacturer’s protocol with the following modifications: the volume of lysis solutions (PartA and PartB) were doubled; an RNase digestion step was performed as suggested in the manufacturer’s protocol; twice the volume of precipitation solution was added; elution was performed using elution buffer EB (Qiagen). A 200 bp genomic library was prepared for isolate *FERA 1166* using a TrueSeq protocol (TrueSeq Kit, Illumina) and sequenced using 76 bp paired-end reads on an Illumina GA2 Genome Analyzer. Genomic libraries were prepared for the other eleven isolates using a Nextera Sample Preparation Kit (Illumina) and libraries sequenced using a MiSeq Benchtop Analyzer (Illumina) using 250 bp, paired-end reads.

RNAseq was performed to aid training of gene models. mRNA was extracted from isolates *FERA 1166* and *FERA 650* grown in full strength PDB, 1% PDB, Potato Carrot Broth (PCB), and V8 juice broth (V8B). The protocol for making PCB and V8B was as described in Simmons (2007) for making Potato Carrot Agar and V8 juice agar, with the exception that agar was not added to the recipe. Cultures were grown in conical flasks containing 250 ml of each liquid medium for 14 days. mRNA extraction was performed on freeze dried mycelium using the RNeasy Plant RNA extraction Kit (Qiagen). Concentration and quality of mRNA samples were assessed using a Bioanalyzer (Agilent Technologies). mRNA from the sample grown in 1% PDB for isolate *FERA 650* showed evidence of degradation and was not used further. Samples were pooled from growth mediums for each isolate and 200 bp cDNA libraries prepared using a TrueSeq Kit (Illumina). These libraries were sequenced in multiplex on a MiSeq (Illumina) using 200 bp paired-end reads.

### Genome Assembly and Annotation

*De novo* genome assembly was performed for all 12 isolates. Assembly for isolate *FERA 650* was generated using SMARTdenovo^1^ (February 26, 2017 github commit), whereas assembly for isolate *FERA 1166* was generated by merging a SMARTdenovo assembly with a MinION-Illumina hybrid SPAdes v.3.9.0 assembly using quickmerge v.0.2 (Antipov et al., 2016; Chakraborty et al., 2016). Prior to assembly, adapters were removed from MinION reads using Porechop v.0.1.0 and reads were further trimmed and corrected using Canu v.1.6 (Ruan, 2016; Koren et al., 2017). Following initial assembly, contigs were corrected using MinION reads through ten rounds of Racon (May 29, 2017 github commit) correction (Vaser et al., 2017)and one round of correction using MinION signal information with nanopolish (v0.9.0) (Loman et al., 2015). Final correction was performed through ten rounds of Pilon v.1.17 (Walker et al., 2014) using Illumina sequence data. Assemblies for the ten isolates with Illumina-only data were generated using SPAdes v.3.9.0 (Bankevich et al., 2012). Assembly quality statistics were summarized using Quast v.4.5 (Gurevich et al., 2013). Single copy core Ascomycete genes were identified within the assembly using BUSCO v.3 and used to assess assembly completeness (Simão et al., 2015). RepeatModeler, RepeatMasker, and TransposonPSI were used to identify repetitive and low complexity regions^2^ ^,3^. Visualization of whole genome alignments between *FERA 1166* and *FERA 650* was performed using circos v.0.6 (Krzywinski et al., 2009), following whole genome alignment using the nucmer tool as part of the MUMmer package v.4.0 (Marçais et al., 2018).

Gene prediction was performed on softmasked genomes using Braker1 v.2 (Hoff et al., 2016), a pipeline for automated training and gene prediction of AUGUSTUS v.3.1 (Stanke and Morgenstern, 2005). Additional gene models were called in intergenic regions using CodingQuarry v.2 (Testa et al., 2015). Braker1 was run using the “fungal” flag and CodingQuarry was run using the “pathogen” flag. RNAseq data generated from *FERA 1166* and *FERA 650* were aligned to each genome using STAR v.2.5.3a (Dobin et al., 2013), and used in the training of Braker1 and CodingQuarry gene models. Orthology was identified between the 12 predicted proteomes using OrthoMCL v.2.0.9 (Li et al., 2003) with an inflation value of 5.

Draft functional genome annotations were determined for gene models using InterProScan-5.18-57.0 (Jones et al., 2014) and through identifying homology (BLASTP, e-value >1 × 10^–100^) between predicted proteins and those contained in the March 2018 release of the SwissProt database (Bairoch and Apweiler, 2000). Putative secreted proteins were identified through prediction of signal peptides using SignalP v.4.1 and removing those predicted to contain transmembrane domains using TMHMM v.2.0 (Käll et al., 2004; Krogh et al., 2001). Additional programs were used to provide evidence of effectors and pathogenicity factors. EffectorP v.1.0 was used to screen secreted proteins for characteristics of length, net charge and amino acid content typical of fungal effectors (Sperschneider et al., 2016). Secreted proteins were also screened for carbohydrate active enzymes using HMMER3 (Mistry et al., 2013) and HMM models from the dbCAN database (Huang et al., 2018). DNA binding domains associated with transcription factors (Shelest, 2017) were identified along with two additional fungal-specific transcription factors domains (IPR007219 and IPR021858). Annotated assemblies were submitted as Whole Genome Shotgun projects to DDBJ/ENA/GenBank (Table 1). This included passing assemblies through the NCBI contamination screen, which did not identify presence of contaminant organisms.

**TABLE 1:** NCBI biosample and genome accession numbers of data generated in this study.

**Isolate**	**BioSample**	**Accession**
*FERA 675*	SAMN06205217	PDUP01000000
*RGR 97.0013*	SAMN06205218	PDWY01000000
*RGR 97.0016*	SAMN06205219	PEJP01000000
*FERA 650*	SAMN06205220	PDWZ02000000
*FERA 1082*	SAMN06205221	PDXA01000000
*FERA 1164*	SAMN06205222	PDXB01000000
*FERA 1166*	SAMN06205223	PDXC01000000
*FERA 1177*	SAMN06205224	PDXD01000000
*FERA 24350*	SAMN06205225	PDXE01000000
*FERA 635*	SAMN06205226	PDXF01000000
*FERA 648*	SAMN06205227	PDXG01000000
*FERA 743*	SAMN06205228	PDXH01000000

### Phylogenetics

BUSCO hits of single copy core ascomycete genes to assemblies were extracted and retained if a single hit was found in all of the 12 sequenced genomes and 23 publicly available *Alternaria* spp. genomes from the *Alternaria* genomes database (Dang et al., 2015). Nucleotide sequences from the resulting hits of 500 loci were aligned using MAFFT v6.864b (Katoh and Standley, 2013), before alignments were trimmed using trimAl v.1.4.1 (Capella-Gutiérrez et al., 2009), and trees calculated for each locus using RAxML v.8.1.17 (Liu et al., 2011). The most parsimonious tree from each RAxML run was used to determine a single consensus phylogeny of the 500 loci using ASTRAL v.5.6.1 (Zhang et al., 2018). The resulting tree was visualized using the R package GGtree v.1.12.4 (Yu et al., 2016).

### CDC Identification

Contigs unique to apple and pear pathotypes were identified through read alignment to assembled genomes. Short read alignment was performed using Bowtie2 (Langmead and Salzberg, 2012), returning a single best alignment for each paired read, whereas long read alignments were performed using Minimap2 v.2.8-r711-dirty (Li, 2018). Read coverage was quantified from these alignments using Samtools (Li et al., 2009).

### Toxin-Synthesis Genes in *Alternaria* Genomes

Sequence data for 40 genes located in *A. alternata* HST gene clusters were downloaded from GenBank. BLASTn searches were performed for all 40 gene sequences against one another to identify homology between these sequences. Genes were considered homologous where they had >70% identical sequences over the entire query length, and an e-value of 1 × 10^–30^. tBLASTx was used to search for the presence of these genes in assemblies.

### Signatures of Genetic Exchange

Mating type idiomorphs present in publicly available genomes were identified using BLASTn searches. A wider assessment within 89 characterized *Alternaria* isolates (Armitage et al., 2015) was undertaken using specific PCR primers (Arie et al., 2000). PCR primers (AAM1-3: 5′-TCCCAAACTCGCAGTG GCAAG-3′; AAM1-3: 5′-GATTACTCTTCTCCGCAGTG-3; M2F: 5′-AAGGCTCCTCGACCGATGAA-3; M2R: 5′-CTGG GAGTATACTTGTAGTC-3) were run in multiplex with PCR reaction mixtures consisting of 10 μl redtaq (REDTaq ReadyMix PCR Reaction Mix, Sigma-Aldrich), 2 μl DNA, 1 μl of each primer (20 μM), and 4 μl purified water (Sigma-Aldrich). PCR reaction conditions comprised of an initial 60 s denaturing step at 94°C followed by 30 cycles of a melting step of 94°C for 30 s, an annealing step at 57°C for 30 s, and an extension step at 72°C for 60 s, these cycles were followed by a final extension step at 72°C for 420 s. MAT1-1-1 or MAT1-2-1 idiomorphs were determined through presence of a 271 or 576 bp product following gel electrophoresis, respectively.

### PCR Screens for Apple and Pear Toxin-Synthesis Genes

A set of 90 previously characterized isolates was used to further investigate the distribution of pathotypes throughout the *A. alternata* species group. PCR primers were designed for the amplification of three genes (*AMT4*, *AMT14*, *AKT3*) located within CDC gene clusters involved in toxin synthesis. Primers for *AMT4* were designed to amplify apple pathotype isolates, *AKT3* to amplify pear pathotype isolates and *AMT14* to identify both apple and pear pathotype isolates. These primers were then used to screen isolates for the presence of these genes in 30 cycles of PCR using 0.25 μl Dream taq, 1 μl of 10x PCR buffer, 1 μl of dNTPs, 1 μl of gDNA, 1 μl of each primer (5 μM), and 4.75 μl purified water (Sigma-Aldrich). PCR products were visualized using gel electrophoresis and amplicon identity confirmed through Sanger sequencing. Primers AMT4-EMR-F (5′-CTCGACGACGGTTTGGAGAA-3) and AMT4-EMR-R (5′-TTCCTTCGCATCAATGCCCT-3) were used for amplification of AMT4. Primers AKT3-EMR-F (5′-GCAATGGACGCAGACGATTC-3) and AKT3-EMR-R (5′- CTTGGAAGCCAGGCCAACTA-3) were used for amplification of *AKT3*. Primers AMT14-EMR-F (5′-TTTCTGCAACGGCG KCGCTT-3) and AMT14-EMR-R (5′-TGAGGAGTYAGACCR GRCGC-3) were used for amplification of *AMT14*. PCR reaction conditions were the same as described above for mating type loci, but with annealing performed at 66°C for all primer pairs.

### Virulence Assay

Pathogenicity assays were performed on apple *cv.* Spartan and *cv.* Bramley’s seedling to determine differences in isolate virulence between *A. tenuissima* isolates possessing the apple pathotype CDC (*FERA 635*, *FERA 743* or *FERA 1166*) and non-pathotype isolates lacking the CDC (*FERA 648*, *FERA 1082* or *FERA 1164*). Briefly, leaves were inoculated with 10 μl of 1 × 10^5^ spore suspensions at six points and the number of leaf spots counted at 14 days post inoculation. One isolate was infected per leaf, with 10 replicates per cultivar. Binomial regression using a generalized linear model (GLM) was used to analyse the number of resulting lesions per leaf.

Unfolded adult apple leaves, less than 10 cm in length were cut from young (less than 12 months old) apple *cv.* Spartan trees or *cv.* Bramley’s seedling trees. These were quality-checked to ensure that they were healthy and free from disease. Leaves were grouped by similar size and age and organized into ten experimental replicates of nine leaves. Leaves placed in clear plastic containers, with the abaxial leaf surface facing upwards. The base of these boxes was lined with two sheets of paper towel, and wetted with 50 ml of sterile distilled water (SDW). The cultivars were assessed in two independent experiments.

Spore suspensions were made by growing *A. alternata* isolates on 1% PDA plates for 4 weeks at 23°C before flooding the plate with 2 ml of SDW, scraping the plate with a disposable L-shaped spreader. Each leaf was inoculated with 10 ml of 1 × 10^5^ spores ml^–1^ *A. alternata* spore suspension or 10 ml of sterile-distilled water at six points on the abaxial leaf surface. Of the nine leaves in each box, three leaves were inoculated with a spore suspensions from isolates carrying apple pathotype CDC, three leaves were inoculated with non-pathotype isolates lacking the CDC, and three leaves were inoculated with SDW. Following inoculation, each container was sealed and placed in plastic bags to prevent moisture loss. Boxes were then kept at 23°C with a 12 h light/12 h dark cycle.

## Results

### Generation of Near-Complete Genomes for the Apple and Pear Pathotype Using MinION Sequencing

**FIGURE 1:**
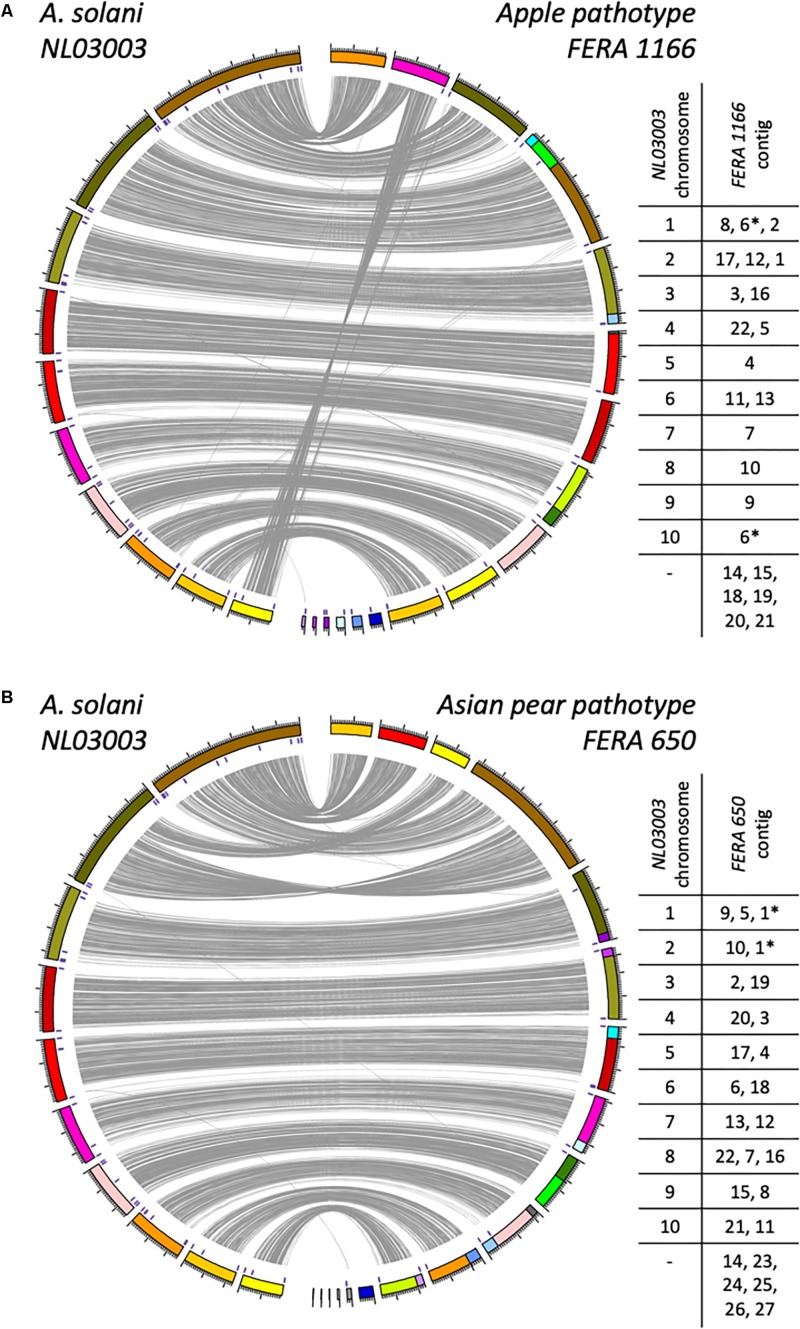
Genome alignment between the reference *A. solani* genome and long-read assemblies of *A. alternata* apple **(A)** and Asian pear **(B)** pathotypes. Links are shown between aligned regions. Locations of telomere repeat sequences are marked within assembled contigs. Contig order in reference to *A. solani* chromosomes is summarized, with those contigs displaying evidence of structural rearrangement marked with an asterisk.

Assemblies using nanopore long-read sequence data for the apple pathotype isolate *FERA 1166*, and pear pathotype isolate *FERA 650* were highly contiguous, with the former totaling 35.7 Mb in 22 contigs and the latter totaling 34.3 Mb in 27 contigs (Table 2). Whole genome alignments of these assemblies to the 10 chromosomes of *A. solani* showed an overall macrosynteny between genomes (Figure 1), but with structural rearrangement of apple pathotype chromosomes in comparison to *A. solani* chromosomes 1 and 10. The Asian pear pathotype had distinct structural rearrangements in comparison to *A. solani*, chromosomes 1 and 2 (Figure 1). Scaffolded contigs of *FERA 1166* spanned the entire length of *A. solani* chromosomes 2, 3, 6, 8, and 9 and chromosomes 4, 5, 6, and 10 for *FERA 650* (Figure 1). Interestingly, sites of major structural rearrangements within *A. solani* chromosome 1 were flanked by telomere-like TTAGGG sequences.

**TABLE 2:** Sequence data, assembly and gene prediction statistics for genomes from three *A. alternata* species group lineages, including apple and Asian pear pathotype isolates.

Organism	*A. gaisen*	*A. tenuissima*								*A. arborescens*		
Pathotype	Pear	Apple				Non-pathotype				Non-pathotype		
Isolate	FERA	FERA	FERA	FERA	FERA	FERA	FERA	FERA	FERA	FERA	RGR	RGR
	650	1166	635	743	1177	648	1082	1164	24350	675	97.0013	97.0016
MinION sequencing depth	34	40	–	–	–	–	–	–	–	–	–	–
Illumina sequencing depth	35	50	27	41	62	54	22	29	37	36	37	28
Assembly size (Mb)	34.3	35.7	36.1	35.9	35.6	33.5	33.9	34.7	33.0	33.9	33.8	33.8
Contigs	27	22	912	788	735	124	345	250	167	325	287	339
Largest contig (Mb)	6.26	3.90	1.43	3.88	4.61	3.50	1.72	2.04	1.90	2.09	1.61	1.06
N50 (kb)	2,110	1,584	319	622	537	1,341	547	709	608	862	685	528
RepeatMasked (kb)	749	1,268	953	869	865	658	628	983	466	853	955	784
RepeatMasked (%)	2.18	3.55	2.64	2.42	2.43	1.96	1.85	2.83	1.41	2.51	2.83	2.32
Conserved Ascomycete genes in genome (%)	98.7	99.0	98.8	98.9	98.8	99.0	98.6	98.8	99.0	98.6	98.6	98.7
Total genes	13169	13576	13733	13707	13580	12757	13028	13114	12806	12896	12766	12820
Total proteins	13220	13633	13812	13776	13647	12798	13091	13169	12856	12936	12813	12863
Conserved Ascomycete genes in gene models (%)	98.7	99.0	98.1	98.2	97.7	98.3	98.2	98.5	98.4	97.7	98.1	98.1
Secreted proteins	1208	1251	1261	1247	1270	1228	1246	1235	1225	1199	1189	1185
Secreted EffectorP proteins	246	248	264	263	268	233	252	252	241	236	229	229
Secreted CAZYme proteins	383	389	397	390	397	392	401	385	391	375	372	382
Secondary metabolite clusters	30	34	36	36	30	28	30	29	26	28	28	30

*Sequencing depth is shown, representing median coverage of trimmed reads aligned to the assembled genome. Number of genes predicted to encode secreted proteins, secreted effectors (EffectorP) and secreted carbohydrate active enzymes (CAZymes) are shown as well as the total number of secondary metabolite clusters in the genome. The percentage of 1315 conserved ascomycete genes that were identified as complete and present in a single copy within assemblies or gene models are shown.*

**FIGURE 2:**
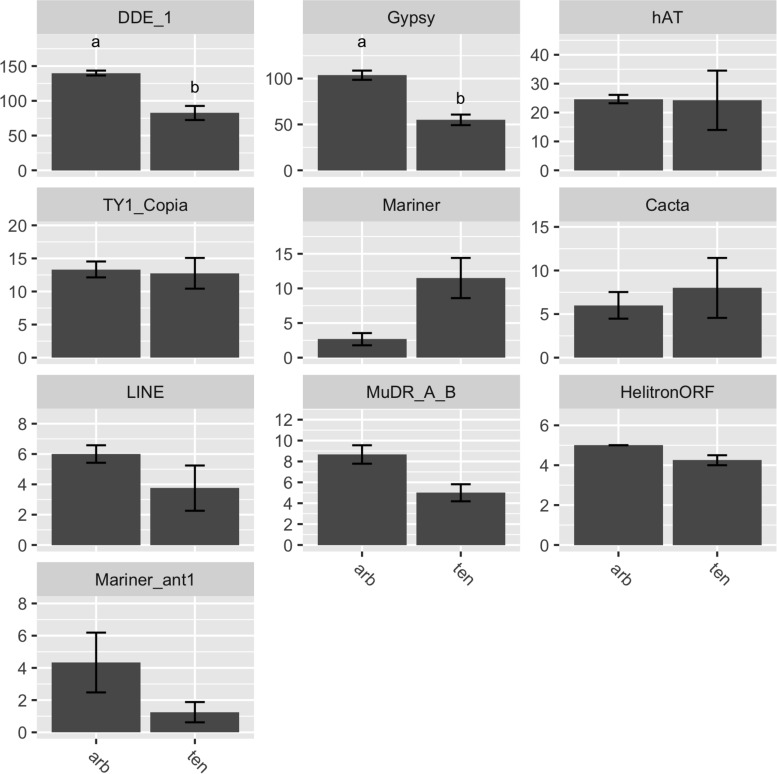
Distinct DDE and gypsy family transposon families between genomes of *A. arborescens* (arb) and *A. tenuissima* (ten) clade isolates. Numbers of identified transposons are also shown for hAT, TY1 copia, mariner, cacta, LINE, MuDR/Mu transposases, helitrons, and the Ant1-like mariner elements.

Genome assembly of 10 Illumina sequenced isolates yielded assemblies of a similar total size to MinION assemblies (33.9–36.1 Mb) but fragmented into 167-912 contigs. Assembled genomes were repeat sparse, with 1.41–2.83% of genomes repeat masked (Table 2). Genome assemblies of *A. arborescens* isolates (33.8–33.9 Mb), were of similar total size to non-pathotype *A. tenuissima* isolates and had similar repetitive content (2.51–2.83 and 1.41–2.83%, respectively). Despite this, identification of transposon families in both genomes showed expansion of DDE (*T*_5__df_ = 5.36, *P* > 0.01) and gypsy (*T*_5__df_ = 6.35, *P* > 0.01) families in *A. arborescens* genomes (Figure 2).

### Phylogeny of Sequenced Isolates

**FIGURE 3:**
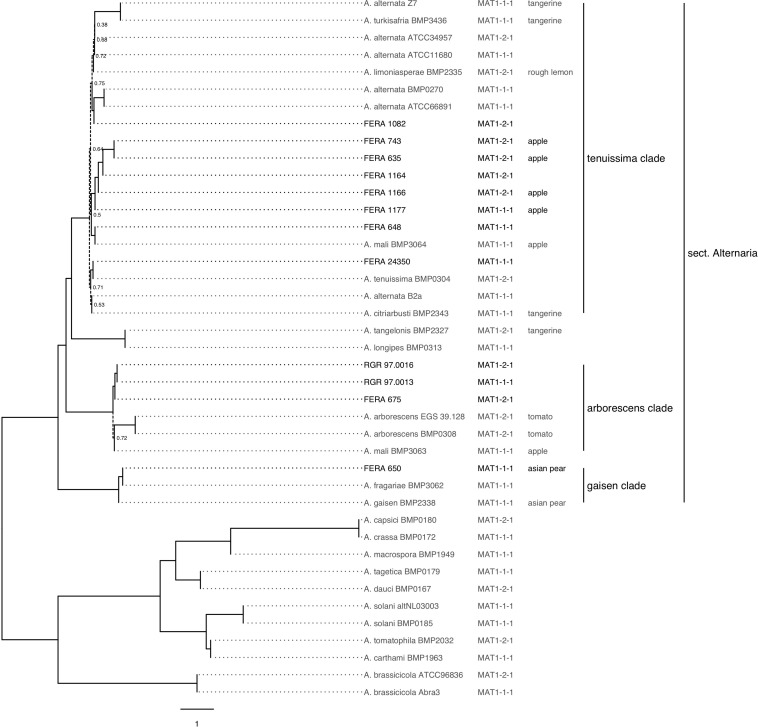
Phylogeny of sequenced and publicly available *Alternaria* spp. genomes. Maximum parsimony consensus phylogeny of 500 conserved single copy loci. Dotted lines show branches with support from <80% of trees. Mating type idiomorphs MAT1-1-1 and MAT1-2-1 show distribution throughout the phylogeny. Isolates pathotype is labeled following identification of genes involved in synthesis of apple, pear, strawberry, tangerine, rough lemon, and tomato toxins.

The relationship between the 12 sequenced isolates and 23 *Alternaria* spp. with publicly available genomes was investigated through phylogenetic analysis of 500 shared core ascomycete genes. *A. pori* and *A. destruens* genomes were excluded from the analysis due to low numbers of complete single copy ascomycete genes being found in their assemblies (Supplementary Table S1). The 12 sequenced isolates were distributed throughout *A. gaisen*, *A. tenuissima*, and *A. arborescens* clades (Figure 3). The resulting phylogeny (Figure 3), formed the basis for later assessment of CDC presence and mating type distribution among newly sequenced and publicly available genomes, as discussed below.

### Gene and Effector Identification

Gene prediction resulted in 12757–13733 genes from the our assemblies (Table 2), with significantly more genes observed in the apple pathotype isolates than in *A. tenuissima* clade non-pathotype isolates (*P* > 0.01, *F*_2_,_8__df_ = 51.19). BUSCO analysis identified that gene models included over 97% of the single copy conserved ascomycete genes, indicating well trained gene models. Apple pathotype isolates possessed greater numbers of secondary metabolite clusters (*P* > 0.01, *F*_2_,_8__df_ = 8.96) and secreted genes (*P* > 0.01, *F*_2_,_8__df_ = 44.21) than non-pathotype *A. tenuissima* isolates, indicating that CDCs contain additional secreted effectors. Non-pathotype *A. tenuissima* clade isolates were found to possess greater numbers of genes encoding secreted proteins than *A. arborescens* isolates (*P* > 0.01, *F*_2_,_8__df_ = 44.21), including secreted CAZYmes (*P* > 0.01, *F*_2_,_8__df_ = 9.83). The basis of differentiation between these taxa was investigated further.

### Genomic Differences Between *A. tenuissima* and *A. arborescens* Clades

Orthology analysis was performed upon the combined set of 158,280 total proteins from the 12 sequenced isolates. In total, 99.2% of proteins clustered into 14,187 orthogroups. Of these, 10,669 orthogroups were shared between all isolates, with 10,016 consisting of a single gene from each isolate. This analysis allowed the identification of 239 orthogroups that were either unique to *A. arborescens* isolates or expanded in comparison to non-pathotype *A. tenuissima* isolates.

Expanded and unique genes to *A. arborescens* isolates was further investigated using *FERA 675* (Supplementary Table S2). Genes involved in reproductive isolation were in this set, including 21 of the 148 heterokaryon incompatibility (HET) loci from *FERA 675*. CAZymes were also identified within this set, three of which showed presence of chitin binding activity and the other three having roles in xylan or pectin degradation. In total, 25 genes encoding secreted proteins were within this set, secreted proteins with pathogenicity-associated functional annotations included a lipase, a chloroperoxidase, an aerolysin-like toxin, a serine protease and an aspartic peptidase. A further six secreted genes had an effector-like structure by EffectorP but no further functional annotations. Furthermore, one gene from this set was predicted to encode a fungal-specific transcription factor unique to *A. arborescens* isolates.

Further to the identification of genes unique or expanded in *A. arborescens*, 220 orthogroups were identified as unique or expanded in the *A. tenuissima*. These orthogroups were further investigated using isolate *FERA 648* (Supplementary Table S2). This set also contained genes involved in reproductive isolation, including nine of the 153 from *FERA 648*. CAZymes within the set included two chitin binding proteins, indicating a divergence of LysM effectors between *A. tenuissima* and *A. arborescens* lineages. The five additional CAZymes in this set represented distinct families from those expanded/unique in *A. arborescens*, including carboxylesterases, chitooligosaccharide oxidase, and sialidase. In total, 18 proteins from this set were predicted as secreted, including proteins with cupin protein domains, leucine rich-repeats, astacin family peptidase domains and with four predicted to have effector-like structures but no further annotations. *A. tenuissima* isolates had their own complement of transcription factors, represented by four genes within this set.

### Identification of CDC Contigs and Assessment of Copy Number

Alignment of Illumina reads to the apple and Asian pear pathotype MinION reference assemblies identified variable presence of some contigs, identifying these as contigs representing CDCs (CDC contigs). Six contigs totaling 1.87 Mb were designated as CDCs in the apple pathotype reference (Table 3) and four contigs totaling 1.47 Mb designated as CDCs in the pear pathotype reference (Table 4). Two *A. tenuissima* clade non-pathotype isolates (*FERA 1082*, *FERA 1164*) were noted to possess apple pathotype CDC contigs 14 and 19 totaling 0.78 Mb (Table 3).

**TABLE 3:** Identification of CDC regions in the *A. alternata* apple pathotype reference genome.

FERA 1166 contig	Length (bp)	*A. gaisen*	*A. tenuissima*								*A. arborescens*		
		Pear	Apple				–				–		
		FERA	FERA	FERA	FERA	FERA	FERA	FERA	FERA	FERA	FERA	RGR	RGR
		650	1166	635	743	1177	648	1082	1164	24350	675	97.0013	97.0016
1	3902980	32	49	26	40	60	53	21	28	36	34	35	26
2	3781932	31	49	26	40	58	51	20	29	35	33	34	26
3	3000390	33	50	26	40	59	53	21	28	36	34	35	26
4	2851745	32	49	27	41	59	53	21	28	36	34	36	27
5	2693844	33	49	27	40	60	53	21	29	36	34	36	27
6	2583941	33	50	27	40	60	53	21	29	36	34	36	27
7	2502671	33	49	26	40	59	52	21	28	36	34	35	26
8	2455819	32	49	27	40	60	52	21	29	35	33	35	26
9	2451092	32	49	26	40	59	52	20	28	35	33	35	26
10	2402550	31	49	26	40	58	51	21	28	35	32	34	25
11	2194253	32	49	26	40	59	52	21	28	35	34	35	26
12	1303685	33	49	27	40	59	53	21	28	36	34	36	27
13	767820	23	48	24	36	47	44	17	25	29	25	26	20
14 (CDC)	549494	0	81	29	79	115	0	23	33	0	0	0	0
15 (CDC)	435297	62	90	51	79	407	0	0	0	0	0	0	0
16	433285	23	48	26	40	53	51	18	28	33	26	27	20
17	391795	26	48	20	33	56	51	19	27	29	30	30	23
18 (CDC)	368761	0	49	22	40	32	0	0	0	0	0	0	0
19 (CDC)	225742	0	48	22	61	72	0	18	31	0	0	0	0
20 (CDC)	154051	0	69	20	45	214	0	0	0	0	0	0	0
21 (CDC)	143777	0	49	20	36	219	0	0	0	0	0	0	0
22	109256	25	50	20	30	50	35	14	22	30	26	27	22

*Read depth is shown from illumina reads against each contig, by isolate. Contigs showing reductions in coverage from non-pathotype isolates were identified as regions of conditionally dispensable chromosomes (CDCs).*

**TABLE 4:** Identification of CDC regions in the *A. alternata* Asian pear pathotype reference genome.

FERA 650 contig	Length (bp)	*A. gaisen*	*A. tenuissima*								*A. arborescens*		
		Pear	Apple				–				–		
		FERA	FERA	FERA	FERA	FERA	FERA	FERA	FERA	FERA	FERA	RGR	RGR
		650	1166	635	743	1177	648	1082	1164	24350	675	97.0013	97.0016
1	6257968	35	43	25	38	58	50	20	28	35	33	34	26
2	2925786	35	45	25	39	58	51	20	28	35	34	35	26
3	2776589	35	44	26	39	58	51	20	28	35	34	35	26
4	2321443	35	45	26	39	58	51	20	28	35	34	35	27
5	2116911	35	46	26	40	59	52	21	29	36	35	36	27
6	2110033	35	44	25	39	58	51	20	28	35	33	35	26
7	1975041	35	42	25	38	56	50	19	27	34	33	34	26
8	1822907	35	43	25	39	58	51	20	28	35	33	35	26
9	1811374	34	42	25	38	57	49	19	27	34	32	34	25
10	1696734	34	42	25	38	56	50	19	27	34	32	34	26
11	1617057	35	43	25	38	57	50	19	27	34	33	34	26
12	1416541	35	47	26	40	60	53	21	29	36	35	36	27
13	1110254	35	39	24	36	55	48	19	26	33	31	32	24
14 (CDC)	629968	68	49	21	41	202	0	0	0	0	0	0	0
15	554254	34	40	24	37	56	48	19	27	33	32	33	25
16 (CDC)	547262	34	8	13	18	22	20	5	14	11	8	8	6
17	522351	35	37	23	35	55	47	18	26	33	31	33	24
18	463030	34	30	22	33	49	44	17	24	29	25	28	21
19	353531	34	30	21	34	49	43	16	23	30	26	28	21
20	350965	34	20	18	27	45	40	15	19	26	22	20	18
21	313768	35	24	19	29	46	38	15	22	27	25	28	19
22	288922	33	35	23	35	51	44	21	24	31	28	30	22
23 (CDC)	206183	34	1	3	5	43	0	0	10	1	0	0	0
24 (CDC)	89891	70	0	0	0	0	0	0	0	0	0	0	0
25	25201	16	0	0	0	0	0	0	0	0	0	0	0
26	23394	5	0	0	0	0	0	0	0	0	0	0	0
27	19592	17	0	0	0	0	0	0	0	0	0	0	0

*Read depth is shown from illumina reads against each contig, by isolate Contigs showing reductions in coverage from non-pathotype isolates were identified as regions of conditionally dispensable chromosomes (CDCs).*

Read alignments showed that CDC contigs were present in multiple copies within *A. alternata* pathotype isolates. *FERA 1166* Illumina reads aligned to its own assembly showed two-fold coverage over contigs 14, 15, 20, and 21 in comparison to core contigs (Table 3). This was more pronounced in isolate *FERA 1177* that had between two- and eight-fold coverage of these contigs. The same was observed in pear pathotype CDC regions, with contigs 14 and 24 in isolate *FERA 650* showing two-fold coverage from Illumina reads in comparison to core contigs (Table 4).

### Toxin Gene Clusters Are Present on Multiple CDC Contigs

Homologs to 15 of the 17 AMT cluster genes were located on contigs 20 and 21 in the apple pathotype reference genome (e-value < 1 × 10^–30^, > 70% query alignment), confirming them as CDC-regions (Table 5). Of the remaining two genes, *AMT11* had low-confidence BLAST homologs on contigs 18 and 21 (e-value < 1 × 10^–30^) whereas the best BLAST hit of *AMT15* was located on contig 18 (e-value < 1 × 10^–30^). Duplication of toxin gene regions was observed between CDC contigs, with contig 20 carrying homologs to 16 toxin genes, but with contig 21 also carrying the *AMT1* to *AMT12* section of the cluster (Table 5). The three other apple pathotype isolates (*FERA 635*, *FERA 743* and *FERA 1177*) also showed presence of 15 of the 17 AMT genes (e-value < 1 × 10^–30^, >70% query alignment), and with some AMT genes present in multiple copies within the genome indicating that the AMT toxin region has also been duplicated in these isolates.

**TABLE 5:** Genomic location (contig number) of homologs to genes from apple, pear, strawberry, tangerine, rough lemon, and tomato toxin gene clusters.

Pathotype	Gene	NCBI Accession	Group	FERA 635	FERA 743	FERA 1166	FERA 1177	FERA 650
Apple	AMT1	AB525198		201,224	146,160	20, 21	81	
	AMT2	AB525198	a	224	160	20, 21	81	
	AMT3	AB525198		224	160	21	81	
	AMT4	AB525198		178	140	20, 21	81	
	AMT5	AB525198		178	140	20, 21	81	
	AMT6	AB525198		178	140	20, 21	81	
	AMT7	AB525198		178	140	20, 21	81	
	AMT8	AB525198		178	140	20, 21	81	
	AMT9	AB525198		178	140	20, 21	81	
	AMT10	AB525198		178	143	20	81	
	AMT11	AB525198		199*	144*	21*	81*	
	AMT12	AB525198		199	144	20	129	
	AMT13	AB525198		200	129	20	128	
	AMT14	AB525198		200	129	20	128	14
	AMT15	AB525198				18**		14*
	AMT16	AB525198		200, 12	129, 13	20, 7	128, 21	12
	AMTR1	AB525198		230	143	20	81	14
Pear	AKT1	AB015351	b					14, 24
	AKT2	AB015352	c					14
	AKT3	AB035492	d					14, 24
	AKTR	AB035491	e					14, 24
Strawberry	AFT1-1	AB070711	b					14, 24
	AFT3-1	AB070713	d					14, 24
	AFT3-2	AB179766	d					14, 24
	AFT9-1	AB179766						14, 24
	AFT10-1	AB179766						14, 24
	AFT11-1	AB179766						14, 24
	AFT12-1	AB179766						24
	AFTS1	AB119280	a	224	160	20, 21	81	
	AFTR-1	AB070712	e					14, 24
	AFTR-2	AB179766	e					14, 24
Tangerine	ACTT1	AB034586	b					14, 24
	ACTT2	AB432914	c					14
	ACTT3	AB176941	d					14, 24
	ACTTR	AB176941	e					14, 24
	ACTT5	AB444613						24
	ACTT6	AB444614						14
Rough Lemon	ACRTS1	AB688098						
	ACRTS2	AB725683						
Tomato	ALT1	AB465676						

Results from reference genome isolates *FERA 1166* and *FERA 650* indicate toxin clusters are present in multiple copies within the genome. This is supported by identification of multiple AMT1 homologs in *FERA 635* and *FERA 743*. Homology between query sequences is shown (homolog groups), as determined from reciprocal BLAST searches between queries. Homologs are identified by e-value < 1 × 10^–30^, >70% query alignment. *Marks lower-confidence hits with <70% query alignment.

The Asian pear pathotype was also found to carry toxin gene clusters in multiple copies, with homologs to the four AKT cluster genes present on contig 14 of the *FERA 650* assembly (e-value <1 × 10^–30^, >70% query alignment), with three of these also present on contig 24 (e-value <1 × 10^–30^, two with >70% query alignment). BLAST hit results from AKT genes were supported by their homologs from strawberry and tangerine pathotypes also found in these regions (Table 5). The pear pathotype genome was also found to contain additional homologs from apple (*AMT14*), strawberry (*AFT9-1*, *AFT10-1*, *AFT11-1*, and *AFT12-1*) and citrus (*ACTT5* and *ACTT6*) located on CDC contigs 14 and 24 (Table 5).

### CDCs Carry Effectors Alongside Secondary Metabolites

A total of 624 proteins were encoded on the six contigs designated as CDCs in the reference apple pathotype genome, with 502 proteins encoded on the four Asian pear pathotype CDC contigs (Supplementary Table S3). We further investigated the gene complements of these regions.

Approximately a quarter of gene models on apple pathotype CDC contigs were involved in secondary metabolism, with 153 genes present in six secondary metabolite gene clusters. This included AMT toxin gene homologs on contigs 20 and 21, which were located within NRPS secondary metabolite gene clusters. Three other secondary metabolite clusters were located on CDC contigs with two of these involved in the production of T1PKS secondary metabolites and the third with unknown function. A further two secondary metabolite clusters were located on contig 14 shared with two non-pathotype isolates, one of which is involved in the production of a T1PKS. The pear pathotype also carried 153 genes in secondary metabolite gene clusters. These 30% of CDC genes were located in four clusters, with the AKT toxin genes in T1PKS clusters of contigs 14 and 24. A second cluster was present on contig 14 with unknown function and a T1PKS cluster was present on contig 16.

Approximately 5% of the genes on apple CDC contigs encoded secreted proteins, with 32 in isolate *FERA 116*6 many of which had potential effector functions with six designated as CAZymes and 12 testing positive by EffectorP. Similarly, a total of 41 secreted proteins were predicted on the CDC regions of the Asian pear pathotype, with eight of these designated as secreted CAZymes and 13 testing positive by EffectorP. Further investigation into the 32 secreted proteins from the apple pathotype identified three CAZYmes from the chitin-active AA11 family, two from the cellulose-active GH61 family and one cellulose-active GH3 family protein. Six of the 13 EffectorP proteins also had domains identifiable by interproscan: four carried NTF2-like domains, which are envelope proteins facilitating protein transport into the nucleus; one was a fungal hydrophobin protein; one was a member of an panther superfamily PTHR40845 that shares structural similarity with proteins from the plant pathogens *Phaeosphaeria nodorum*, *Sclerotinia sclerotiorum*, and *Ustilago maydis*. Of the 38 secreted proteins identified from the pear pathotype, two CAZYmes were also identified from the chitin-active AA11 family, two from the AA3 family with single proteins from GH5, CBM67 and AA7 families. Ten of the twelve secreted EffectorP proteins had no functional information as predicted by interproscan, with the other two identified as carrying WSC domains IPR002889, which are cysteine-rich domains involved carbohydrate binding. CDCs may also play important roles in transcriptional regulation with 29 putative transcription factors identified in the apple pathotype CDC contigs (4.6% CDC genes) and 35 identified in pear pathotype CDC contigs (7.0% CDC genes).

### Polyphyletic Distribution of Apple and Tangerine Pathotypes

The evolutionary relationship between *A. alternata* pathotypes sequenced in this study and publicly available genomes was analyzed by the core gene phylogeny (Figure 2). We identified four isolates as tangerine pathotypes (Z7, BMP2343, BMP2327, BMP3436) two as tomato pathotypes (BMP0308, EGS39-128), one Asian pear pathotype (MBP2338), one rough-lemon (BMP2335) and two apple pathotypes (BMP3063, BMP3064) through searches for genes from HST-gene clusters (Supplementary Table S4). When plotted on the genome phylogeny, we found the apple and tangerine pathotypes to be polyphyletic (Figure 2). Five of the six sequenced apple pathotype isolates were located in the *A. tenuissima* clade and one in the *A. arborescens* clade, whereas the tangerine pathotype was present in both the *A. tenuissima* clade and in the *A. tangelonis/A. longipes* clade.

### Molecular Tools for Identification of Apple, Pear, and Strawberry Pathotypes

**FIGURE 4:**
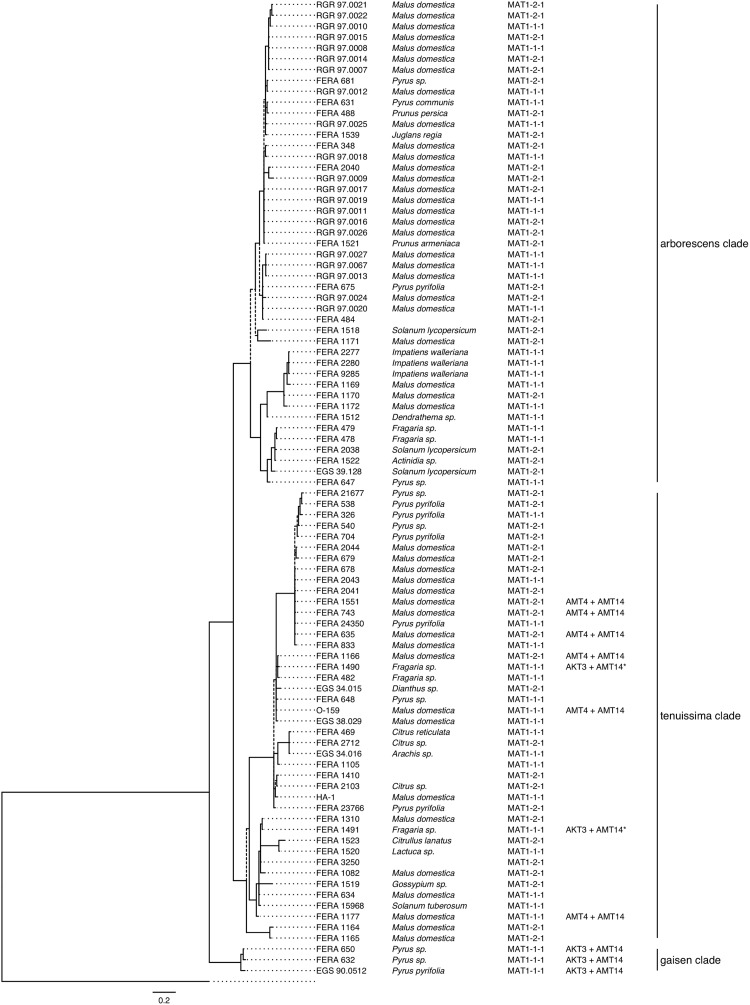
Presence/absence of toxin and mating type genes for 89 *Alternaria* isolates. Results are plotted onto the 5-gene phylogeny of Armitage et al. (2015). MAT1-1-1 and MAT1-2-1 mating type idiomorphs are designated.

PCR primers for three loci (*AMT4*, *AKT3*, and *AMT14*) were designed to identify the distribution of pathotypic isolates through the *A. alternata* species group and were screened against a set of 89 previously characterized isolates (Figure 4). Five isolates tested positive for the presence of *AMT4*, each of which was from the *A. tenuissima* clade (*FERA 635*, *FERA 743*, *FERA 1166*, *FERA 1177*). Five isolates tested positive for the presence of *AKT3*, including the three isolates from Asian pear in the *A. gaisen* clade and a further two isolates from the *A. tenuissima* clade that were from strawberry. Sequencing of the *AKT3* amplicons from the two isolates *ex.* strawberry identified them as the *AFT3-2* ortholog of *AKT3*, showing that these isolates were strawberry pathotypes rather than pear pathotypes. Sequencing of PCR products from the other isolates confirmed them to be apple or pear pathotypes as expected. All of the isolates testing positive for *AMT4* or *AKT3* also tested positive for *AMT14*, indicating its suitability as a target gene for identification of a range of pathotypes.

**FIGURE 5:**
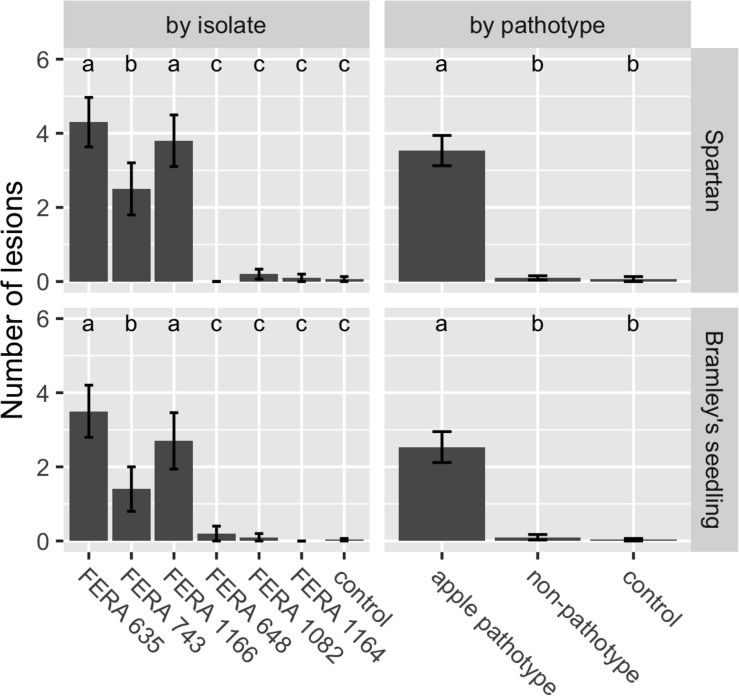
Number of *Alternaria* lesions per apple leaf at 14 dpi for treatments in virulence assays on *cv.* Spartan or *cv.* Bramley’s Seedling leaves. Apple pathotype isolates (*FERA 635*, *FERA 743*, and *FERA 1166*) cause significant disease symptoms in comparison to control leaves, in contrast to non-pathotype isolates (*FERA 648*, *FERA 1082*, and *FERA 1164*). Number of lesions (±SE) are shown with significance at *P* < 0.05, as determined from a GLM at the isolate and pathotype level.

Presence of apple pathotype CDCs was confirmed to be associated with pathogenicity through detached apple leaf assays. Apple pathotype isolates showed significantly greater numbers of necrotic lesions when inoculated onto *cv.* Spartan (*F*_72__df_ = 100.64) and *cv.* Bramley’s Seedling (*F*_72__df_ = 69.64) leaves than non-pathotype *A. tenuissima* isolates (Figure 5).

### Signatures of Genetic Exchange

Of the 12 sequenced isolates, BLAST searches identified five as carrying the MAT1-1-1 idiomorph and seven as carrying MAT1-2-1. Both idiomorphs showed distribution throughout the *A. alternata* genome phylogeny (Figure 2). These results were supported by PCR assays identifying the mating type of 89 previously characterized isolates (Figure 4). Idiomorphs did not deviate from a 1:1 ratio within *A. tenuissima* (21 MAT1-1-1: 23 MAT1-2-1; χ^2^ = 0.09, 1df, *P* > 0.05) or *A. arborescens* clades (18 MAT1-1-1: 24 MAT1-2-1; χ^2^ = 0.86; 1df; *P* > 0.05), as expected under a random mating population. All three of the *A. gaisen* clade isolates carried the MAT1-2-1 idiomorph.

## Discussion

This work builds upon the current genomic resources available for *Alternaria*, including the *A. brassicicola* and *A. solani* genomes (Belmas et al., 2018; Wolters et al., 2018), *A. alternata* from onion (Bihon et al., 2016) the additional 25 *Alternaria* spp. genomes available on the *Alternaria* Genomes Database (Dang et al., 2015) as well as recent genomes for other pathotype and non-pathotype *A. alternata* (Hou et al., 2016; Wang et al., 2016; Nguyen et al., 2016). Of the previously sequenced genomes, *A. solani*, the citrus pathotype and a non-pathotype *A. alternata* isolate have benefited from long read sequencing technology with each comprising less than 30 contigs (Wolters et al., 2018; Wang et al., 2016; Nguyen et al., 2016). Total genome sizes in this study (33–36 Mb) were in line with previous estimates for *A. alternata*, with the tomato pathotype also previously assembled into 34 Mb (Hu et al., 2012). Synteny analysis of our two reference genomes against the chromosome-level *A. solani* genome revealed structural differences for chromosomes 1 and 10 in the apple pathotype and for chromosomes 1 and 2 in the pear pathotype. These structural differences may represent distinct traits between clades of the *A. alternata* species group, and may represent a barrier to genetic exchange involved in the divergence of *A. gaisen* and *A. tenuissima* lineages. The number of essential chromosomes in our reference genomes is in line with previous findings in *A. alternata* (Kodama et al., 1998), with 9-11 core.

Species designations within the species group have been subject to recent revision (Woudenberg et al., 2015; Lawrence et al., 2013; Armitage et al., 2015) leading to potential confusion when selecting isolates for study. For example, the available *Alternaria fragariae* genome (Dang et al., 2015), did not represent a strawberry pathotype isolate and was located in the *A. gaisen* clade. As such, the phylogenetic context for sequenced *Alternaria* genomes described in this study, along with pathotype identification provides a useful framework for isolate selection in future work.

### Evidence of Genetic Exchange

A 1:1 ratio of MAT loci was observed within *A. arborescens* and *A. tenuissima* clades. This supports previous identification of both idiomorphs within *A. alternata*, *Alternaria brassicae*, and *A. brassicicola* (Berbee et al., 2003). Furthermore, presence of both MAT idiomorphs within apple pathotype isolates indicates that genetic exchange (sexuality or parasexuality) has occurred since the evolution of CDCs, providing a mechanism of transfer of CDCs. Evidence for cryptic sexuality or a parasexual cycle has been previously presented for the citrus pathotype of *Alternaria alternata* (Stewart et al., 2013). We also show that some recent or historic genetic exchange has occurred between *A. tenuissima* and *A. arborescens* clades, with both apple and tangerine pathotypes exhibiting a polyphyletic distribution throughout the phylogeny.

### Duplication of Toxin-Gene Contigs

Toxin genes have been proposed to be present in multiple copies within *A.* sect. *alternaria* pathotype genomes with *AMT2* proposed to be present in at least three copies in the apple pathotype CDC (Harimoto et al., 2008), and multiple copies of *AKTR* and *AKT3* in the pear pathotype (Tanaka et al., 1999; Tanaka and Tsuge, 2000). Through read mapping we demonstrated that this is the case. Furthermore, we show that toxin gene clusters are present on multiple contigs, with differences in the gene complements between these clusters. At this stage, it is unclear whether these different clusters are responsible for the production of the variant R-groups previously characterized in AMT or AKT toxins (Nakashima et al., 1985; Harimoto et al., 2007). Differences were also noted between non-pathotype isolates from the *A. tenuissima* clade in the presence/absence of contigs 14 and 19, representing a total of 775 kb. Chromosomal loss has been reported in the apple pathotype (Johnson et al., 2001), and it is not clear if this represents chromosomal instability in culture or additional dispensable chromosomes within *A. tenuissima* clade isolates.

### PCR Primers for Diagnostics

It is now clear that genes on essential chromosomes do not provide reliable targets for identification of different pathotypes and hence loci located directly on CDCs should be used. We found *AMT14* homologs to be present in all pathotype genomes and designed primers to this region. These demonstrated specificity to apple, pear and strawberry pathotypes within a set of 86 *Alternaria* isolates. Furthermore, Sanger sequencing of these amplicons confirmed this to be a single locus that can both identify and discriminate a range of pathotypes. Wider validation of this primer set is now required to test its suitability across other pathotypes.

### Divergence of *A. arborescens* and *A. tenuissima*

The divergence of *A. tenuissima* and *A. arborescens* lineages was investigated through identification of expanded and unique gene compliments. We identified HET loci unique to *A. arborescens* or *A. tenuissima* lineages. HET loci may act as incompatibility barriers to common genetic exchange between these taxa (Glass and Kaneko, 2003). Taxa also showed divergence in effector profiles, including chitin binding effectors, with *A. arborescens* isolates possessing unique xylan/pectin degradation CAZymes, while *A. tenuissima* isolates possessed unique carboxylesterase, chitooligosaccharide and sialidase CAZymes. Chitin binding proteins are important in preventing MAMP triggered host recognition by plants and animals during infection, and may also aid persistence of resting bodies outside of the host (Kombrink and Thomma, 2013). Putative transcription factors were also amongst the proteins specific to *A. arborescens* or *A. tenuissima*, indicating that these taxa not only possess distinct gene complements but also differ in how they respond to stimuli. Dispersed repeat sequences such as transposable elements have been shown to serve as sites of recombination within and between fungal chromosomes (Zolan, 1995) and we also show distinct transposon profiles between *A. arborescens* and *A. tenuissima*. Transposons are known to aid host adaptation in plant pathogens (Faino et al., 2016; Gijzen, 2009; Schmidt et al., 2013) and have been a mechanism for differentiation of these taxa.

### Effectors on CDC Regions

*Alternaria* HSTs are capable of inducing necrosis on non-host leaves (Kohmoto et al., 1976), meaning that non-host resistance must be associated with recognition of other avirulence genes. We investigated the complements of other putative pathogenicity genes and effectors produced by the apple and Asian pear pathotypes and identified additional CAZymes and secondary metabolite profiles on CDC regions, distinct between pathotypes, suggesting additional host-adapted tools for pathogenicity. Additional secondary metabolites clusters were present on both apple and pear pathotype CDCs as well as unique complements of secreted CAZymes. CAZyme families AA3, AA7 and AA9 have previously been reported to be in greater numbers in the citrus pathotype in comparison to non-pathotypes (Wang et al., 2016). Furthermore, putative transcription factor genes were identified in CDCs indicating that these regions may have some level of transcriptional autonomy from the core genome. This has been shown in *Fusarium*, where effector proteins are regulated by the SGE transcription factor on the core genome but also by FTF and other transcription factor families (TF1-9) located on lineage specific chromosomes (van der Does et al., 2016).

## Conclusion

We report near-complete reference genomes for the apple and Asian pear pathotypes of *A.* sect. *alternaria* and provide genomic resources for a further ten diverse isolates from this clade. For the first time we show sequenced *Alternaria* genomes in a phylogenetic context allowing the identification of both mating type idiomorphs present in *A. arborescens* and *A. tenuissima*, with a distribution throughout subclades that was indicative of recent genetic exchange. The presence of the apple CDC in isolates of both mating types supports gene flow between isolates. Furthermore, the distribution of isolates from different pathotypes throughout the phylogeny indicated that apple and tangerine pathotypes are polyphyletic. This means that gene flow is not limited to within, but has also occurred between *A. tenuissima* and *A. arborescens* lineages. We also developed PCR primers to aid identification of pathotypes, with those targeting the AMT14 locus identifying a range of pathotypes due to its conservation between CDCs. Despite evidence of genetic exchange between *A. arborescens* and *A. tenuissima* clades, we show that these taxa are sufficiently isolated to have diverged, with significant differences in core effector profiles and transposon content.

## Data Availability Statement

Accession numbers for genomic data are provided in Table 1. Sanger sequence data is deposited on NCBI under accession numbers MK255031–MK255052.

## Author Contributions

AA, SS, JW, CL, and JC contributed to the conception and design of the study. AA, HC, and RH performed the lab work including library preparation and sequencing. AA performed the bioinformatic analyses and wrote the manuscript. All authors contributed to the manuscript revision, read, and approved the submitted version.

## Conflict of Interest

CL was employed by FERA Science Ltd. The remaining authors declare that the research was conducted in the absence of any commercial or financial relationships that could be construed as a potential conflict of interest.

## Footnotes

1 https://github.com/ruanjue/smartdenovo

2 http://www.repeatmasker.org

3 http://transposonpsi.sourceforge.net

## Supplementary Material

TABLE S1 Identification of single copy Ascomycete genes in reference *Alternaria* spp. genomes.

TABLE S2 Gene IDs, location, and functional annotations of genes in expanded orthogroups between *A. tenuissima* and *A. arborescens*.

TABLE S3 Gene IDs, location and functional annotations of genes located on CDC contigs from apple and Asian pear pathotype isolates.

TABLE S4 Toxin gene BLAST hits in publicly available *Alternaria* spp. genomes, allowing identification of pathotype isolates.
